# Staff perspectives of barriers to women accessing birthing services in Nepal: a qualitative study

**DOI:** 10.1186/s12884-015-0564-6

**Published:** 2015-07-02

**Authors:** Lesley Milne, Edwin van Teijlingen, Vanora Hundley, Padam Simkhada, Jillian Ireland

**Affiliations:** Centre for Midwifery, Maternal & Perinatal Health, Faculty of Health and Social Science, Bournemouth University, Dorset, UK; Centre for Public Health, Liverpool John Moores University, Liverpool, UK; Poole Foundation NHS Trust, Dorset, UK

**Keywords:** Skilled birth attendant, Health personnel, Barriers, Access, Childbirth, Maternity, South Asia

## Abstract

**Background:**

Nepal has made significant progress with regard to reducing the maternal mortality ratio but a major challenge remains the under-utilisation of skilled birth attendants who are predominantly facility based. Studies have explored women’s views of the barriers to facility birth; however the voices of staff who offer services have not been studied in detail. This research explores the views of staff as to the key reasons why pregnant women do not give birth in a maternity-care facility.

**Methods:**

This mixed methods study comprised qualitative interviews and non-participant observation. The study was conducted in two small non-governmental hospitals, one semi-rural and one urban, in Kathmandu Valley. Twenty interviews were conducted with health care providers and other staff in these hospitals. The interviews were undertaken with the aid of a Nepali translator, with some interviews being held in English. Twenty-five hours of non-participant observation was conducted in both maternity hospitals . Both observation and interview data were analysed thematically. Ethical approval was granted by the Nepal Research Health Council and Bournemouth University’s Ethics Committee.

**Results:**

Key themes that emerged from the analysis reflected barriers that women experience in accessing services at different conceptual levels and resembled the three phases of delay model by Thaddeus and Maine. This framework is used to present the barriers. First Phase Delays are: 1) lack of awareness that the facility/services exist; 2) women being too busy to attend; 3) poor services; 4) embarrassment; and 5) financial issues. Themes for the second Phase of Delay are: 1) birthing on the way; and 2) by-passing the facility in favour of one further away. The final Phase involved: 1) absence of an enabling environment; and 2) disrespectful care.

**Conclusion:**

This study highlights a multitude of barriers, not all of the same importance or occuring at the same time in the pregnancy journey. It is clear that staff are aware of many of the barriers for women in reaching the facility to give birth, and these fit with previous literature of women’s views. However, staff had limited insight into barriers occuring within the facility itself and were more likely to suggest that this was a problem for other institutions and not theirs.

## Background

Reduction of maternal mortality is one of the key Millennium Development Goals (MDGs) and is measured by two indicators: a reduction in the maternal mortality ratio (MMR) by three quarters and the proportion of deliveries attended by a skilled health person [1]. Nepal has made significant progress with regard to the first of these indicators, reducing the MMR from 539 per 100,000 live births in 2006 (one of the highest in the world) [2] to 170 per 100,000 in 2010 [3]. Possible explanation for this decline include: the rapid drop in total fertility, increase in average age of marriage, the 2002 Abortion law, and the high proportion of Nepali men working abroad [4–7]. However, one of the major challenges remains the underutilisation of Skilled Birth Attendants (SBA) and the health facilities where they invariably practise [8].

Traditional barriers to facility birth in low resource settings include costs, transportation problems, and sociocultural norms [9], and a lack of necessary infrastructure, equipment, supplies, drugs and systems for referral that comprise an enabling environment [10]. However, recent literature points to staff behaviour as a significant deterrent to women entering facilities [11]. Research conducted in Nepal suggests that poor quality of services, unavailability, and inaccessibility of SBAs, minimal staff support, lack of medicine and equipment and poor referral systems lead to a low uptake of skilled attendance at birth [12, 13]. Other constraints to providing effective maternal health services include staff knowledge and competence, lack of proper training and development, inadequate pay, and lack of support from management and colleagues [14]. The cost of maternity care in an institution, either real or perceived, is also a factor affecting the uptake of facility birth [15–19].

Previous studies have explored women’s views of these barriers to facility birth. Recently, Morrison et al. [19] interviewed women who had birthed at home in rural Nepal to explore their reasons for delay in seeking care (the ‘first delay’) and concluded that decisions were based on a mix of considerations. These included a lack of family support around the time of birth, difficulty in securing funding for transport, the thought of shaming their family by displaying their body parts in the facility, and past poor encounters. Women’s accounts and experiences provide valuable data in the search for strategies to overcome the barriers to accessing skilled care, however further information from the provider perspective is needed. Although it is recognised that women’s voices from marginal communities are seldom heard, the voices of staff who offer services to these women have had even less recognition in the literature. Reducing barriers to facility based care requires not only a commitment from health care providers but also an understanding of their perspectives and awareness if practices are to be changed to improve care for women. This study sought to elicit the views of health care providers in two maternity units in Nepal, one semi-rural (SR) and one urban (U), regarding barriers to facility birth. Acknowledging the value of the staff’s first-hand experience not only adds to the available evidence but can determine possible improvements for service provision if any deficits are highlighted.

## Methods

This mixed methods study comprised 20 qualitative interviews with ten members of staff in each birthing facilty and 25 h of non-participant observation in both facilites. Written informed consent was ontained from all praticipants. A comprehensive literature search was conducted to place the study within the wider body of knowledge.

### Literature search

The Advanced Search, with no time restriction, was conducted on MySearch, a federated search engine, provided by EBSCO. Databases covered included Global Health, MEDLINE Complete, Science Citation Index, Social Sciences Citation Index, CINAHL, ScienceDirect, PsycINFO, guided by a librarian. Medical Subject Headings (MESH) and key words included SBA OR Skilled Birth Attendan* (truncation), health personnel or obstetrician* or gyn*cologist* or p*diatrician* or nurs* or midwi* AND developing countr*AND “point* of view*” or perspective* AND Childbirth NOT “wom*n* perspective*” Due to resources the search was limited to English language papers only.

### Setting

The study was conducted in one not-for-profit semi-rural community hospital in Kathmandu Valley (hospital SR) and one small private urban hospital (hospital U), in Kathmandu. These hospitals were chosen because they are community hospitals dealing with low risk women from poorer communities. The hospitals represent the type of facilities that the government of Nepal is currently advocating to increase facility birth. In hospital SR all members of staff work full time, usually forty hours per week without night shift or 48 h with a night shift. Nursing staff included axillary-nurse midwives, community medical assistants and health promoters who are supported by receptionists and cleaners for example. There is only one general doctor on site covering 24 h a day, seven days a week. There is no paediatrician, obstetrician or gynaecologist, however every Saturday morning one female obstetric gynaecologist (ObGy) is scheduled to come from Kathmandu city to conduct a gynaecology clinic that includes an antenatal clinic. Hospital SR only has provision for normal vaginal deliveries; they do not have the equipment, facilities or trained personnel to support caesarean sections or assisted deliveries. In addition, they do not have paediatric cover or intensive care facilities to receive and care for sick babies. The hospital provides an on call ambulance manned by two ambulance drivers covering a 24 h, seven day a week service. Neither drivers have had any first aid nor other health related training.

In contrast Hospital U, established 18 months prior to data collection, is located near a major and extremely busy intersection in Kathmandu. The hospital is open 24 h a day, seven days a week. The facilities include a four-bedded antenatal room where women also labour, a delivery room that can accommodate one birth at a time, and a separate four bedded ward that doubles as both a postnatal ward and mixed-sex medical/surgical ward. There is also an operating theatre. Additional services available between 4 and 6 o’clock in the evenings include obstetric, paediatric and anaesthetic cover but there are no intensive care facilities. There is no ambulance service.

### Data collection

Data were collected through one-to-one, face-to-face, semi-structured interviews with health care providers, conducted over a period of one month in two hospitals (September 2013). Non-participant observation was used to provide further insights into the barriers portrayed by the health care providers.

A purposive sample of staff was interviewed on each site. Staff ranged from SBAs, including auxiliary nurse midwives and doctors, to support staff such as laboratory technicians and receptionists. Questions for the semi-structured interviews were developed in English and were guided, in part, by themes emerging from the literature around barriers from women’s perspectives. The questions were pre-tested on members of the research team in English. Once translated by a native Nepali speaker, questions were further tested and reviewed by three Nepali colleagues and edited accordingly prior to the interviews being conducted. Interviews were conducted in English, where possible, with those staff whose English was good enough, in order to reduce any bias arising through interpretation [20]. A Nepali interpreter who had received all her schooling in English was used for non-English speaking staff. Interviews were audio-taped (with permission) and, where necessary, translated before being transcribed. Four interviews in Nepali were transcribed twice by separate translators for quality control.

Birth registers were reviewed to determine how many women used the facility for birth and the outcomes for both mothers and babies. Both sites were able to produce evidence from their respective birth registers covering the last 12 months up to the point of the research taking place.

Non-participant observations of women and staff interacting in both facilities were also undertaken; however few interactions were witnessed due to low numbers of labouring women accessing either facility. All kinds of staff interactions were observed, and no members of staff objected to being observed. These observations were captured through field notes taken as events occurred or shortly afterwards. Additional observations were undertaken at an antenatal clinic delivered on site at Hospital SR and an outreach antenatal clinic provided by staff from Hospital U.

A thematic approach to analysing the qualitative data was used [21]. Three researchers (LM, JI and EvT) coded all of the transcripts independently. Emergent themes were then discussed and agreed and during this process it became apparent that key themes corresponded with Thaddeus and Maine’s three Phases of Delay so quotations, extracted from participants’ (P) transcripts, are presented using this framework to illustrate themes [22].

#### Ethical considerations

Ethical approval was given from both the Nepal Health Research Council and Bournemouth University’s ethics committee. Each participant received a study information sheet, written in Nepali assuring that confidentiality would be maintained.

## Results

### Literature search

**Fig. 1:**
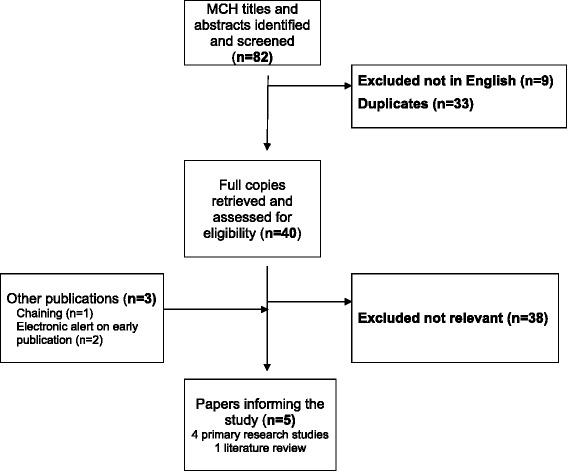
Search and selection process

The search strategy initially yielded 82 results, 73 were written in English and after duplicates were removed 40 results remained. After reviewing these by title and abstract, 38 were regarded as irrelevant because they did not focus on the perspectives of health care providers, leaving two articles (Fig. 1) [23, 24]. Articles were also identified through backward chaining authors’ relevant, personal collections [25] and a further 2 [19, 26] recently acquired by electronic article alerts. The original search was re-run prior to publication and no further articles were identified.

Blum et al. [23] compared the experiences of 13 SBAs who facilitated births in both home and hospital settings in rural Bangladesh. From the SBAs’ perspectives facility birth provided an environment more favourable to effective skilled attendance than home birth.

Kawuwa et al. [24] sought to identify ten service providers’ perspectives on barriers to addressing obstetric complications quickly and effectively in a Nigerian district hospital in which they worked. All participants agreed that many women arrived at the facility in a poor state of health and provision of care was then hindered by a lack of appropriately skilled health personnel, equipment, drugs and supplies. They conclude that participants were also aware of the main barriers causing delays for women at both community and facility level echoing findings from Thaddeus and Maine [22]. However, this short paper does not offer any qualitative insight into the issues raised.

In northern Ethiopia, health carers percieved transport difficulties and their own lack of ability together with indequately resourced working environments to be key barriers to women accessing their services [26].

Nepalese studies used a variety of qualitative methods of data collection and were undertaken as components of larger scale mix method studies that detail either the qualitative findings from users’ perspectives [27], quantitative findings also from user’s perspectives [17, 28] or both [29]. Only one study was solely qualitative and this explored women’s reasons for home birth [19]. There were no studies reporting qualitative findings from staff perspectives.

In addition to the four primary papers, one paper [30] reviewed the literature on both women’s and staff’s perceptions. This review of the available international literature (published till 2010) explored factors affecting women’s uptake of skilled birth attendants for birth and then discussed these with particular reference to Nepal. The findings, drawn predominantly from quantitative research, reiterated findings from other countries. Their work also highlighted the lack of qualitative research detailing how and why these factors are responsible. The literature review confirmed the lack of research around SBAs’ and other hospital staff’s perceptions of barriers to women accessing their services.

### Interviews

The 20 staff that were interviewed (ten in each hospital) included support staff (n = 4), auxiliary nurse-midwives (n = 3), community medical assistants/health assistants (n = 4), staff nurse-midwives (n = 5) medical officer/obstetric gynaecologist (n = 2), and other health staff (n = 2).

**Fig. 2:**
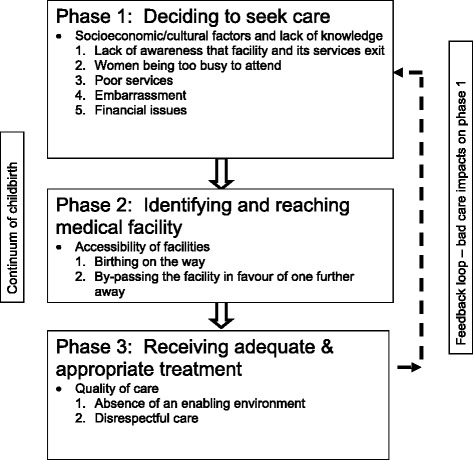
Adaptation of the three Phases of Delay model [22]

The quotes obtained from participants are presented within themes using Thaddeus and Maine’s three Phases of Delay as a framework [22]. Figure 2 highlights the key barriers to women accessing their services during the three phases of delay.

#### First phase of delay

This phase centres on delays to seeking care [22]. Key themes are: 1) lack of awareness that the facility and its services exist; 2) women being too busy to attend; 3) poor services; 4) embarrassment; and 5) financial issues.

1. Lack of awareness that the facility and its services exist
  Several participants believed that some women failed to access services because they were uneducated and participants assumed therefore that some women were unaware of the facility’s existence or the importance of being attended by a SBA during childbirth. Others suggested that, whilst women may know that the hospital exists they do not see its value or are unaware of what it offers and prefer to follow the cultural practice of homebirth. This was particularly noted by some participants working at Hospital U
  “…those who did not acquire education…they do not know about birthing…hospital…” (Hospital U, P1).
  “…our community isn’t so much educated…sometimes they have their previous baby delivered at home and they don’t think it’s important, sometimes…they don’t know about the services…” (Hospital U, P2).
  “…most of the women don’t know what are the advantages of hospital delivery. They are satisfied at home delivery…women’s mother and grandmother delivered child at home and many women still want to follow the same tradition” (Hospital U, P3).
  Participants from both hospitals reported that they attempted to address the perceived lack of community awareness of the facility and services by conducting a variety of hospital-led initiatives
  “Sometimes we are pamphleting” (Hospital U, R3).
  “We do mic’ing (use a microphone) in the community about the available services in this hospital” (Hospital SR, P9).
  “…sometimes they will…conduct a camp… they will say to the community here we have this sort of services in our hospital” (Hospital SR, P1).
  However, the sporadic provision of such initiatives was evident at both sites and several participants from both hospitals commented that these initiatives did not always increase facility use.
2. Women being too busy to attend
  Other participants believed a key barrier was the fact that some women were too busy to attend,
  “…they [women] look after all the house woman stuff, they cook the morning meal, they have to farm the cattle’s…reach the kids going to school…and cook the meal again…” (Hospital U, P10).
  “…they don’t have time to come to hospital when they are giving birth” (Hospital U, P1).”
3. Poor services
  Non-participant observations of interactions between staff and women were undertaken at the weekly antenatal clinics (ANC) at hospital SR. On three consecutive Saturdays women were observed sitting quietly at the clinic alongside staff waiting for the non-arrival of the gynaecologist by taxi from Kathmandu. Comments from participants suggested this was a regular occurrence that they believe affected some women’s future attendance for both antenatal clinic and more specifically, for birth. Observations made of birth registers belonging to both facilities evidenced an average of 5 births in the semi-rural facility and 7 births in the urban facility a month.
  “…nowadays due to problem of gynaecology doctor [not arriving] because they are coming from KTM [Kathmandu] and …there is an obstruction in regular ANC [antenatal] clinic and due to this delivery is also decreasing. Before it was 10–18 deliveries per month now…[for last] 2–3 months…only five delivery per month” (Hospital SR, P4).
  Similar incidents were also reported at hospital U
  “…gynaecologist is available only after 4 pm. In this situation they [women] prefer to go to another hospital” (Hospital U, P3).
  Respondents at hospital SR noted,
  “Some come here for two or three times and go to Kathmandu for final check-up… If 10 are coming for ANC, among them 5 are coming [here] for delivery” (Hospital SR, P5).
  A further respondent at the same hospital estimated that only 25 % of women who attended the facility based antenatal clinic returned for a facility birth (Hospital SR, P1).
  Participants acknowledged that some women who had experienced a facility birth did not continue to access the facility for future pregnancies and birth following a perceived poor experience. One respondent said
  “Some of the women they have had a bad experience…they might not feel comfortable coming here for the next pregnancy” (Hospital U, P10).
  Participants from both facilities also reported that service users ‘word of mouth’ influenced other potential user’s decisions around whether or not to access local facility birth saying
  “…if one of them comes here for delivery she goes back home and says something to her friends about it” (Hospital U, P2).
  Moreover, it was believed that some women had relayed their previous poor experiences to their communities and this had coloured community members’ views which had caused a decline in facility birth at hospital SR. Several participants retold a particular birth story in detail:
  “…delivery was for prolonged labour… after delivery the baby…cried and we had rubbed the baby’s back but instance the baby did not cry we got a little respiration rate…immediately we refer the baby [to a] better place and… after that we got the problem…They [family]claim that we drop the baby in the bucket… after two or three days baby was dead but actually it was not the problem and so the case dismiss…until one month of that case delivery will not come this hospital…she is also from the same community so if she walk along her home…she was also there in the delivery room and she has also dropped baby in the bucket they tell her they complain” (Hospital SR, P1).
4. Embarrassment
  Despite the majority of participants on both sites saying that they would advise their family members and friends to access the facility in which they work for childbirth, two participants in the semi-rural facility chose to give birth in Kathmandu. Both participants were employed to work closely with communities in their catchment area and with the hospital staff. One of their main remits was to encourage women to use their local birthing centre, however they themselves confessed to by-passing the service during their own childbirth due to perceived embarrassment of revealing their body parts to fellow colleagues.
  “…staff who working here, they are coming from same community and they feel the people [staff members of hospital] can see my body’s private part. It makes me shame….They feel embarrassed” (Hospital SR, P10).
  Moreover, participants noted that some women also feel embarrassed about using their local facility for childbirth if they know staff who work there and reside in their community.
  “On past, [Staff member] did delivery to one woman here [hospital] the women thinks nowadays she saw my private part and I can’t see her because of shyness. Community people says like that” (Hospital SR, P10).
  “A number of women feel shy as they don’t want to show…genital area in the hospital” (Hospital U, P6) remarked another respondent.
5. Financial factors
  Some participants believed financial costs deterred many women from accessing their local birthing facility. For example
  “If she is earning herself then she will lose a day’s salary coming here…she have to explain her money for travelling here…” (Hospital U, P10).
  “…[husband] does not provide money for transport…others may not have money for food…during their stay in hospital (Hospital U, P6).
  Conversely, participants also believed that financial incentives were the key reasons why some women accessed their services and participants were well versed in these incentives.
  “In our hospital, we offer 25 % off…blood investigations…as well as USG [ultrasound] abdomen service…For the people who don’t have any money, we told them some of our facilities including free delivery, free bed facilities, thousand rupees incentives and baby clothes free” (Hospital U, P3).
  “…the Government of Nepal…have established the…birthing unit…and wherever they have established those birthing units…whenever women go to the ANC and the labour and after delivery… if they survive … they get thousand rupees…if [they deliver in the elevated flatlands and hills region]” (Hospital U, P10).
  Several participants intimated that most women using the facilities were poorer women who could not afford the travel costs of going to a larger facility and who lived nearby to the birthing centre.
  “…the poor person does not want to go to Kathmandu because they have not money” (Hospital SR, P7).
  “The women coming from far distance may have difficulties because of not having transport but the women who are close from this hospital they don’t have any difficulties” (Hospital SR, P6).

#### Second phase of delay

There was evidence of two themes falling within the second phase of delay, which centres around accessing facilities [22]. The themes are; 1) birthing on the way; and 2) by-passing facility in favour of one further away.

1. Delivering on the way
  It appears usual, for some women not to reach the facility in time to give birth especially during the night. Participants from both sites reported this to be the case.
  “…sometimes… they get the labour pains during the night time and waiting for the vehicle to come to the hospital…she delivers either in the house… or on the way to hospital…[in] the taxi or ambulance and retain the placenta [inside the mother] and come in bleeding and state of shock…we do receive lots of patients like that” (Hospital U, P10).
  “Most of cases, it is not really home delivery. The delivery is conducted in the Ambulance…while coming to hospital. As compared to past, the number of home delivery has gone down these days” (Hospital SR, P5).
  Furthermore, participants from hospital SR reported that no one from the hospital accompanies the ambulance driver to pick the woman up and bring her back to the facility, thus increasing risks to both the mother and baby associated with birth before arrival. This report was also supported by observation.
2. By-passing facility in favour of one further away
  Staff believed that some women circumnavigate their local birthing centre in favour of one or other of the main government maternity hospitals in Kathmandu:
  “Rather than going as referred from here they go directly there” (Hospital SR, P5).
  Some interviewees in the semi-rural centre spoke of women by-passing the birthing centre in favour of what they described as “better” services even if, at that time, the woman had no apparent obstetric complication that warranted referral.
  “…they will go if the cases are normal, they…will go to Kathmandu hospital during the time of delivery or after birth for child complications may come. So they wanted to go to hospital for better treatment” (Hospital SR, P4).
  Moreover, it would appear that some women and their families are in a position to make choices with regard to where they go for birth and SBAs are supporting them in their choices.
  “It also depends on their choices; they can go wherever they want to go…If they say they want to go to [Kathmandu]…it is okay…” (Hospital SR, P5).
  Furthermore, participants demonstrated insight into why they believed some women and their families chose to by-pass their local birth centre and offered a number of explanations that echoed their own beliefs about the services they wished to deliver, rather than the services that they felt they were actually able to deliver.
  “She [the respondent] will not tell her relatives to come here because all the facilities are not here and … if she has severe case she have to go to Kathmandu or other places she will say better to go to other places than come here. Everything is looked by general doctor …[the community] are thinking, oh you are working in that…hospital and it’s not giving enough services for us and we have to go different places…” (Hospital SR, P1).

#### Third phase of delay

Obtaining acceptable, appropriate and timely care which constitutes the final phase of delay [22] gives rise to two further themes that staff identified as barriers to women accessing facility birth. These are: 1). absence of an enabling environment; and 2). Disrespectful care. This particular phase was the phase most likely to be witnessed during the observation element of the study.

1. Absence of an enabling environment
  This theme relates to availability of emergency obstetric and newborn care services including specialist staff, SBAs, infrastructure, equipment, drugs and a clean environment. One respondent commented,
  “Facilities are essential in private hospital but we don’t have. People come for delivery but we have frequent electric cut. So, they dislike here. People complain in this issue…” (Hospital U, P 9).
  During observations of both facilities daily power cuts, which could last several hours at a time, were observed.
  Another respondent suggested,
  “…it [the services] can be improved if the staff get skill birth attendance training, new equipment and can handle a complicated case…caesarean section, breech delivery. And if a women needs to go to operation theatre a specialised gynaecologist doctor will take over” (Hospital SR, P4).
  Whilst only one respondent noted that women expect the facility to be “…clean and tidy” (Hospital U, P8), another respondent commented on the importance of reducing cross infection saying,
  “We have separate room for delivery. CAC (Comprehensive Abortion Care) and PAC (Post Abortion Care) is happening in the same place. We don’t have separate room for that…there may be infection…and…could transfer to babies…There is no procedure for infection prevention…” (Hospital SR, P5).
  Despite the presence of domestic staff carrying out their duties, observations of both hospitals illustrated the lack of adequate facilities to maintain cleanliness. Frequently these were beyond the staff members’ control, such as the intermittent loss of water for washing hands and surfaces.
2. Disrespectful care
  Observations made in antenatal clinic at the outreach post linked to hospital U showed the norm to be a queue of six women waiting to be seen all sharing the same room whilst taking it in turns to be weighed, palpated and have personal questions answered. The antenatal room doubled as the waiting room and consequently women stood in a line waiting their turn as they watched those before them being weighed and examined abdominally. None seemed to be phased by this, observations showed they supported each other holding hands coming into the room together, waiting for each other, interpreting for each other, joining in with questions and answers and then leaving together. There was a sense of community spirit and support. Furthermore, no disrespectful behaviour was observed during observation sessions in both facilities however most of the time the facility was devoid of childbearing women.
  On discussing issues around disrespectful care in hospital settings all participants were able to identify behaviours representative of what they perceived constituted both respectful and disrespectful care giving. A number of participants confirmed they had witnessed disrespectful care but most made it clear that they had not witnessed this behaviour in the facility in which they currently worked.
  “…in some places in Nepali…hospitals nurses will be angry if they [women] cry”. (Hospital SR, P1)
  One respondent also said that the “irregular/unreliable doctor. Not arriving…in time” (Hospital U, P8) constituted disrespectful care.
  Another respondent (Hospital SR, P7) spoke of women being ignored by staff in their local facility and how she then advocated on their behalf and urged her colleagues to deliver better care.

## Discussion

The three phases of delay emerged from the data rather than being used to tailor pre-determined interview questions. The interviews highlight a number of key barriers to all phases of delay that are common to both sample sites, despite their different geographical locations, and that resonate with the literature predominantly collated from women’s perspectives [10, 11, 18, 19]. Less commonly reported themes included poor services, embarrassment and financial issues related to the most recently employed financial incentives [31] (first Phase Delay); by-passing the facility in favour of one further away (second Phase Delay) and both absence of an enabling environment and disrespectful care (third Phase Delay).

Several barriers, such as poor services, were linked to several different phases of delay. For example, participants believed some decisions to access care were influenced by women’s previous poor experiences with their local facility or by stories told by others who had used the facilities and recounted poor services during the third delay. Thus influencing other people’s decisions to either birth at home (the first delay) or by-pass facilities and go elsewhere for subsequent births (the second delay). We know that pregnant women are often influenced by the experiences and stories of friends and family, however in Nepal the mother-in-law and husband tend to be the most influential people [8]. Ingrained cultural factors are very difficult to change and there is a need for greater awareness and solutions that focus on cultural competence rather than change. For example, Nepal might be encouraged to train more female doctors in obstetrics and services encouraged to employ them.

A number of factors appear likely explanations to why women by-pass their local birthing facility in favour of government maternity hospitals further away. A practice initially identified with ill people in Africa [32]. Research conducted in rural Tanzania [33] recognised women frequently by-pass both their local antenatal and birthing facility in favour of services offered at higher level facilities further away regardless of the cost and effort involved in accessing them. Women in Kruk et al.’s study perceived quality of care to be poorer and staff to be less trustworthy at their local facility. Similarly, participants in our study noted that some women having initially accessed local facility antenatal care went on to complete the remainder of their antenatal care and birth in a tertiary hospital in the capital. It was not possible from our study to determine the reasons women had for doing this; however staff hypothesised that it was to do with the greater availability of services and equipment in Kathmandu. Knowing the staff in the local hospital and being embarrassed was also suggested to be a reason for by-passing the service.

What was evident from observations and interviews was the absence of the ObGy in hospital SR and the limited availability of the ObGy in hospital U. However, one very practical issue in both hospitals is that ObGyn doctors also undertake fulltime work elsewhere in main government birthing facilities in Kathmandu and regularly do not turn up for scheduled clinics. This practise is likely to be a result of the lack of qualified ObGy’s nationally coupled with the opportunity to subsidise incomes [34]. Moreover, participants perceived many women had time constraints due to competing demands of daily family life, house chores and working in agriculture. It is possible that the lack of emergency obstetric care locally means people think ahead and plan to go directly to larger facilities in Kathmandu which they may perceive to offer more reliable services assuming they have the money for transport and other associated out of pocket expenses.

Further to this staff relayed a major incident at hospital SR and were aware that stories of this event had been circulated amongst community members and possibly contributed to by-passing which in turn influenced a reduction in their monthly birth numbers.

A notable number of quotes also highlight the use of the term “better care/treatment” used by staff to describe larger tertiary facilities and the use of such descriptors when talking to women and their families may also encourage women to by-pass. In addition both staff and communities’ philosophy on what constitutes safe birth and quality services perhaps focus on a more medicalised model of care and the opportunity to get caesarean section.

As previously mentioned, embarrassment was a key finding. It has been reported in the literature that Nepali women feel shy about allowing others to examine their bodies and is tied in with women’s status and their duty not to bring shame on their family (Morrison et al. 2014). This notion of being embarrassed to show private parts to doctors has also been reported as a barrier to accessing local medical care by Nepalese female sex industry workers [35]. Moreover, several respondents at hospital SR also professed to birthing in a main facility in Kathmandu rather than in the facility that they worked at due to embarrassment and role modelling this ‘flight’ may have also influenced other women to do the same.

Reasons underpinning the decision to by-pass have been postulated. It may be a reflection on perceived poor local services or seemingly better services in the city and further research is needed to ascertain this and to determine to what extent either of these views contribute to the decision and who makes the decision. Having said this, it is clear that there are women who can afford the one hour taxi fare from their semi-rural residence (half an hour from hospital U) to a facility further away. Therefore an unknown proportion of women presumably birth with a SBA albeit at another facility and are disputably exercising freedom of choice.

The effects on by-passing for both the local facilities and tertiary hospitals necessitate further consideration. If the trend to by-pass continues local facilities may be in danger of becoming unviable whilst tertiary staff and facilities, already purported to be overstretched and under-resourced, may find it increasingly difficult to provide adequate care.

One possible solution for the inconsistency of specialist doctor availability for antenatal care is to train midwives adequately to care for low risk women and refer women with risk factors to a reliable tertiary service however this necessitates a reliable ambulance service for transfers and the availability of a SBA for escort.

Financial issues have long been established as a major barrier to accessing SBA care. The Government’s Safe Motherhood Programme, Aama Aurakshya Karyakram, was implemented to alleviate this and offers women 500 rupees in the Terai (plains) and 1,500 rupees in the mountains if they birth in a facility. Concurrently, health facilities receive 1000 rupees (US $11) for a normal birth, 3,000 rupees (US $34) for managing obstetric complications and 7,000 rupees (US $80) per caesarean section [36]. In addition, having a caesarean section at Hospital U necessitates women and their families to purchase pharmaceutical supplies from the neighbouring private pharmacy at a cost of 4,834 rupees (US $46). In contrast Government tertiary hospitals also offer free caesarean section in addition to the same financial incentives as local birthing units and this could be a further factor influencing by-passing.

Barriers within the hospital were less obvious to staff. Although staff highlighted a number of absent environmental effects necessary for them to perform their duties adequately, one major observation noted was the lack of a clean working environment. Only one respondent mentioned the importance and difficulties in maintaining hygiene. It is possible that no other participants highlighted cleanliness as an issue because this reflects the standard facility norm. There is evidence that for a proportion of rural women, standards of cleanliness experienced in government hospitals is higher than where they are required to give birth at home as settings include cowsheds, in keeping with the tradition of ‘chaupadi’ [37]. Whether cleanliness is *perceived* to be better in the tertiary hospitals compared to the local facility is unclear, although a recent study suggests otherwise [38].

Prevention of cross infection is crucial to the working environment, yet observations showed the current circumstances to be incongruent with the idea of promoting safe birth in a facility with a SBA and the belief that this would reduce maternal death will be impeded if cleanliness and the prevention of cross infection are not addressed adequately. In other words, women birthing in facilities may reduce the incidence of primary postpartum haemorrhage for example one of the current major direct cause of maternal death in Nepal [39], but this may be at the expense of increasing the likelihood of women dying later, following discharge, from a hospital acquired infection [40] or indeed a secondary postpartum haemorrhage triggered by infection.

A further observation was the lack of privacy observed at the urban hospital’s antenatal outreach clinic which may be construed in some cultures as disrespectful but in this particular context no pregnant woman appeared phased by this. What may constitute disrespectful behaviour in one culture may be perceived as acceptable, respectful behaviour in another [11]. Staff were aware of disrespectful care occurring but not in their facility and none was observed during the study, although it is possible that staff were ‘performing’ for the observer and the lack of child birthing women meant few interactions were witnessed. Having said this, discussion around disrespect care led some staff to identify that they themselves felt disrespected at times by service users. A recent study carried out in two major government maternity hospitals in Kathmandu focused on female nurse SBAs concepts of respectful maternity care reported that they understood the concept of disrespectful care, but because they were overworked they placed women’s safety over comfort as a priority [41]. Release of the WHO statement on the ‘prevention and elimination of disrespect and abuse during facility-based childbirth’ recognizes the problem and “calls for greater co-operation among governments, healthcare providers, managers, professional associations, researchers, women’s advocates, international organizations and women themselves to end disrespect and abuse during facility-based childbirth” [42].

### Limitations and strengths of study

The scale of this study did not permit the elicitation of women’s and their families’ perceptions which may or may not corroborate with the perceptions of the staff however a number of the findings are supported by both the primary literature review and observations undertaken during the study. A strength of the study is that it did not set out with any preconceived ideas about the barriers from health providers’ perspectives, but it was surprising how well the themes that emerged mapped to the model proposed by Thaddeus and Maine [22].

One weakness is the use of a translator during some interviews as meanings and accuracy of information may be lost, although the same translator was used throughout providing consistency. Accuracy of translation was verified by two Nepalese translators transcribing four of the interviews independently of each other. On the positive side, using a translator enabled access to the wider staff body whom all have a role to play in providing childbirth services and thus influencing women’s perceptions.

A further strength of this study was the concurrent use of observations and semi structured interviews. Whether observations should be conducted prior to the interviews or vice versa is debatable however, entering into the ‘natural world’ and the unpredictability of the nature of this world with regards to the workload of staff for a relatively short period of time necessitated a pragmatic approach and opportunities to interview staff and undertake observations were taken as they arose. Consequently, all participants were interviewed during their working day at their convenience. On several occasions this meant staff members were interviewed one after another, as a result, staff were unable to confer with each other although responses to questions were very similar and thus credible.

## Conclusion

Acknowledging the value of the staff’s first-hand experience not only adds to the available evidence but highlights staffs’ awareness of deficits in service provision that create a number of barriers to women accessing their services for childbirth. This study highlights a multitude of barriers, not all of the same importance or occuring at the same time in the pregnancy journey. It is clear that staff are aware of many of the barriers for women in reaching the facility to give birth, and these fit with previous literature of women’s views. However, staff had limited insight into barriers occuring within the facility itself and were more likely to suggest that this was a problem for other institutions and not theirs.

## Abbreviations

ANC: Antenatal clinic
ObGy: Obstetric gynaecologist
P: Participant
SBA: Skilled birth attendant
SR: Semi-rural
U: Urban
